# Practical solutions for implementation of *Transition to Practice* curricula in a competency-based medical education model.

**DOI:** 10.36834/cmej.67821

**Published:** 2020-08-06

**Authors:** Layli Sanaee, Marla Nayer, Susan Glover Takahashi

**Affiliations:** 1 Department of Medicine, Division of Emergency Medicine, University of Toronto, Ontario, Canada.; 2 University Health Network, Ontario, Canada.; 3 Department of Postgraduate Medical Education, University of Toronto, Ontario, Canada.

## Abstract

**Background:**

Although transition from residency to practice represents a critical learning stage, there is a paucity of literature to inform local curriculum development and implementation.

**Objectives:**

To describe local curriculum development for *Transition to Practice* (TTP) for use within a competency-based medical education model, including important content and suitable teaching and assessment strategies.

**Design:**

We reviewed the literature to construct a definition and develop initial curriculum content for TTP. We then gathered local residency program directors’ views on TTP content, teaching, and assessment via online survey and an international educational conference workshop.

**Results:**

We identified 21 important TTP content areas in the literature and analyzed 35 survey responses, representing 33 residency programs. Survey participants viewed *Further sophistication of clinical skills, How to set up a practice*, and *Time management skills* as the three most important content areas. Views on content importance varied by program. For teaching and assessment strategies, most respondents preferred: assessing what residents could do, providing real-life practice opportunities, and offering workplace-based assessments.

**Conclusions:**

TTP curricula implementation should reflect nationally set, specialty-specific curriculum elements; locally developed priority content; and assessment and teaching strategies. Individual learner needs and imminent practice context should guide faculty approaches to curriculum delivery.

Résumé

## Abstract (fr)

**Contexte:**

Bien que la transition de la résidence à la pratique représente une étape d’apprentissage essentielle, on retrouve peu de littérature pour informer le développement et l’implantation des cursus locaux.

**Objectifs:**

Décrire le développement d’un cursus local de *Transition vers la pratique* (TVP) pour un modèle d’éducation médicale par compétences, incluant le contenu important et des stratégies d’enseignement et d’évaluation adaptées

**Conception:**

Nous avons recensé les écrits afin d’établir une définition et de développer le contenu initial du cursus de TVP. Nous avons ensuite recueilli l’opinion des directions de programmes de résidence locaux sur le contenu, l’enseignement et l’évaluation du cursus TVP par le biais d’un sondage en ligne et un atelier offert dans le cadre d’une conférence pédagogique internationale.

**Résultats:**

Nous avons identifié 21 sujets importants pour la TVP dans la littérature et nous avons analysé les réponses de 35 sondages représentant 33 programmes de résidence. Les trois sujets considérés les plus importants par les participants au sondage étaient le *Perfectionnement avancé des compétences cliniques*; *Comment débuter sa pratique médicale et les Aptitudes de gestion du temps*. Les opinions sur l’importance du contenu variaient selon les programmes. Pour ce qui est des stratégies d’apprentissage et d’enseignement, la plupart des personnes sondées ont exprimé les préférences suivantes : évaluer ce que les résidents sont en mesure de faire; fournir des occasions réelles de pratiquer ; offrir des évaluations en milieu de travail.

**Conclusions:**

La mise en œuvre des cursus TVP devrait refléter des éléments des curriculums propres aux spécialités et définis à l’échelle nationale; des sujets prioritaires développés localement; des stratégies d’enseignement et d’évaluation. Les besoins personnels des étudiants et le contexte imminent du début de pratique devraient orienter les approches du corps professoral utilise dans l’enseignement de ce cursus.

## Introduction

Readiness to practice is an essential professional milestone frequently discussed among medical educators, clinician teachers, and learners, yet not adequately described to sufficiently inform local curriculum development, implementation, and delivery. In view of its significance, the Royal College of Physicians and Surgeons of Canada (RC) has identified *Transition to Practice* (TTP) as a distinct stage of specialty education in its competency-based medical education model, called Competence by Design (CBD). Medical education within this model is conceptualized as a competence continuum, with each stage representing a new phase of learning commensurate with the needs and demonstrated abilities of residents.^1^ Education is outcome-oriented; time is just one of many resources for acquiring competencies.^1^

The four stages of specialty residency education in CBD are:

1. Transition to Discipline
2. Foundations of Discipline
3. Core of Discipline
4. Transition to Practice

Generally, RC examinations in CBD will be moved from the end of residency to *Core of Discipline*, and certification will be granted after the completion of *Transition to Practice*.^1^ The *Transition to Practice* stage comprises entrustable professional activities (EPAs) that shape its curriculum, including content, teaching activities, and assessment tools.^1^^-^^3^

By “curriculum” we mean the objectives or outcomes, teaching and learning content, and teaching paradigms or learning science concepts used. Curriculum work includes five key components: curriculum design, development, implementation, delivery, and evaluation.^4^ These components are connected to each other, and the curriculum process is cyclical, interdependent, and iterative in nature, rather than linear and static.^4^

**Figure 1:**
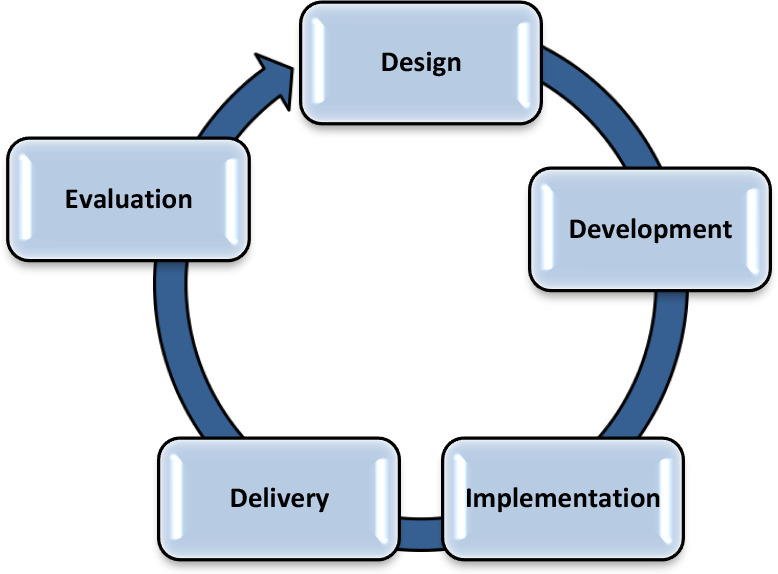
Five key components of curriculum work ^a^ Adapted from: Figure 3, Lazor J., Glover Takahashi S., Leslie K., 2018, 'Who's connected to whom and how: a model of evolving relationships and roles in faculty development and curriculum development during curriculum renewal and innovation', Med Ed Publish, 7, [2], 51,

With guidance from RC education leaders, specialty committees work on curriculum design using national requirements consistent with the CBD model, including EPAs, training experiences, and CanMEDS competencies.^5^ CBD builds on the work of ten Cate, who introduced EPAs as a method of operationalizing competencies into daily clinical practice.^6^^,^^7^ He described EPAs as “units of professional practice,” defining them as “tasks or responsibilities to be entrusted to the unsupervised execution by a trainee once he or she has attained specific competence” ^6^^,^^7^ In the context of specialty medical education, each EPA comprises a set of competencies and can cover several CanMEDS roles.^2^^,^^5^^,^^8^

The curriculum development, implementation, and delivery that follows CBD specialty-specific curriculum design are locally led activities about where, when, and how the speciality requirements will be achieved.^5^ The local experience of developing, implementing and delivering CBD curricula can, in turn, be evaluated to provide feedback to specialty committees on their initial curriculum design of specialty CBD requirements.

There is currently an absence of resources to effectively support local programs in curriculum development, implementation, and delivery of TTP since few programs have residents in the fourth stage of CBD. Given the significance of this stage for success in professional practice, we view it as important to begin curriculum development (i.e. content, teaching strategies, and assessment approaches) of TTP- designed content (e.g. EPAs, educational experiences, and competencies) and to consider how it should be implemented and delivered locally. For example, local programs may consider integrating the TTP design with past curriculum approaches, such as structured Transition to Practice programs,^9^^,^^10^ Junior Attending^a^ experiences, and Chief Resident roles. Studies exploring the perspectives of recent graduates also provide insights for TTP curriculum development priorities.^9^^,^^12^^-^^15^ Examining EPAs for TTP from similar specialties within CBD is also valuable. Arguably of fundamental importance, however, for local programs, clinician educators, and Program Directors, will be developing a greater understanding of the purpose of TTP, in order to frame its EPAs and ultimately plan its curriculum. Once professional activities to be included in the *Transition to Practice* stage are selected, local programs may find it helpful to use Miller’s Framework for Clinical Assessment^16^ (or modified versions^17^) as a starting point to deliberate on corresponding teaching activities and assessment approaches.^5^^,^^16^ Miller’s original framework describes four levels of assessment: knows, knows how, shows how, and does.^16^ One modified version of Miller’s pyramid is a 3-dimentional model that includes Knowledge, Skills, and Attitudes, as well as assessment tools that could be used for six levels of assessment.^17^ This modified framework supports faculty in considering assessment in an expanded manner. For example, how to assess cognition (e.g. knows and knows how, using multiple-choice questions) versus how to assess behaviour (e.g. shows how and does, using simulation and direct observation).

Regardless of medical education model (e.g. competence-based, time-based, or hybrid), readiness to practice is a critical consideration in the curriculum. There is a need for a closer examination of this key transition stage, particularly in the context of CBD curriculum design, as it is under-studied. At the International Conference on Residency Education (ICRE) in 2018, for example, only five of 200 abstracts referred to TTP, and the authors of this paper offered the sole workshop on the topic, out of a total of 105 workshops.^18^ RC publications will help guide specialty committees in establishing EPA curriculum design requirements for TTP; but content is not sufficient for locally relevant curriculum development, implementation, and delivery. This paper provides information for locally relevant curriculum development, implementation, and delivery for TTP, using the views of local Program Directors gathered through an online survey.

The purpose of this paper is to describe locally relevant curriculum development for *Transition to Practice* (TTP), for use within a competency-based medical education model, including the development of important content, as well as suitable teaching and assessment strategies.

## Methods

This paper had five key steps: 1) literature review, 2) survey question design and pilot testing, 3) survey launch, 4) analysis of results, and 5) knowledge mobilization and triangulation from an international conference workshop into manuscript preparation.

For the literature review we had the guidance of a library information specialist. We conducted a search of *Ovid MEDLINE* ® in October 2016, using index terms (MeSH) and keywords for the concepts of residents, transition, and independent practice. We used the following search terms: “internship and residency”, Hospital Medical Staff, transition, career mobility, and career ladder. The search was iterative and included mini-searches related to three associated but distinct concepts: peer mentorship, simulation training, and medical students. We chose these terms to identify relevant literature that may have been missed from the initial strategy. “Peer mentorship” and “simulation training” represent interventions from the literature to smooth the transition to practice. We included “medical students” to explore possible shared themes between *Transition to Discipline* and TTP. In addition, we consulted experts in the field for article suggestions, reviewed reference lists of included articles, screened white papers from Canada and Australia, and reviewed RC publications and ICRE conference abstracts. A single reviewer (LS) assessed articles for consideration, based on the presence of any description of TTP, its purpose, or curriculum content, in the abstract. We considered English language articles and conducted follow-up searches in October 2018 to identify new literature and ICRE conference abstracts.

We compared EPAs for TTP from three residency programs at the University of Toronto (Emergency Medicine, Medical Oncology, and Urology) against the content list to ensure no large domain was missing. These programs were selected due to EPA availability at the time. All specialty committees will eventually set national EPA requirements for TTP in Canada; however, at the start of this study some local programs had identified EPAs for TTP during CBD implementation. Two medical educators outside of the core authorship group, at the University of Toronto, reviewed the content list and tested the survey questions prior to finalization.

Thematic saturation was important for the understanding of content to include in the survey. We collected until no new patterns or themes emerged from the data. In finalizing the content list, we were informed, but not constrained, by the literature. We used CanMEDS language as our anchor, as it is the framework in which CBD operates. For example, although *Quality assurance* and *Quality improvement* are different concepts, only *Quality improvement* was selected for inclusion. Similarly, although there is overlap between *Patient safety, Quality improvement*, and *Patient complication analysis*, they were included as separate items, as they cover different competencies.

We sequenced the survey questions based on Schwab’s model of curriculum development, which involves deliberative consideration of the learners, teachers, content, and context of the best local solution, all things considered.^19^^-^^22^ The working definition and content list were provided to the Program Directors (PDs) as a reference point to inform their survey decisions. We sent the online survey to all 80 PDs, representing 68 specialties and subspecialties, at the University of Toronto, through the Postgraduate Medical Education Office. We included one response per program (the PD, where available), and only fully completed responses in the final analysis. For the 68 Royal College specialties, the University of Toronto has 74 accredited residency programs and six Areas of Focused Competence (AFC) programs, with several of the specialties having both a pediatric program and an adult program. In this paper, we use the terms residency program and specialty interchangeably.

This study is not within the scope of Research Ethics Board (REB) review at the University of Toronto, due to its primary purpose of program evaluation.^23^ They gave is an exemption from a formal review. In accordance with established ethical standards, we explicitly informed survey participants of the voluntary nature of participation and of data confidentiality.^24^ Participants had the option to maintain anonymity by not providing their name or program.

We asked survey respondents to do five things: (1) Identify important content for TTP from a selected list (For your specialty, please identify all that you think are important); (2) Choose their top three content options for TTP (Of the following topics that you identified as important, which are the top three most important?); (3) Identify the corresponding desired outcomes (For your selected priority topic of [field inserted], please indicate the option that best identifies the desired outcome); (4) Identify the preferred corresponding teaching activities (For your selected priority topic of [field inserted], please indicate the option that best identifies the teaching approach); and (5) Identify the preferred corresponding assessment approaches (For your selected priority topic of [field inserted], please indicate the option that best identifies the assessment approach), using a modified version of *Miller’s Framework for Clinical Assessment*.^17^ Appendix A in Supplemental Materials provides a copy of the entire survey. Appendix B includes the Framework for Curriculum Design, Learning, Teaching, and Assessment used in the survey.

Supplementary Tables

## Results

### Defining features, working definition, and content of TTP

This section highlights specific characteristics of the *Transition to Practice* stage, informed by RC publications, current literature, consensus discussion, and previous survey results from University of Toronto PDs.^23^

*Transition to Practice* marks the final stage of specialty residency education. Certain professional activities will be emphasized during this time, with others newly introduced to increase practice readiness. Building on prior stages, residents in TTP enhance their ability to function autonomously and build their confidence to provide quality care for large numbers of patients.^25^ Designed to have an optimal balance of autonomy and accessible supervision, TTP lets learners refine their abilities and cultivate a lifelong orientation towards critical reflection.^3^^,^^26^ Learners are provided the opportunity to apply knowledge and experience, in clinical and team contexts, and to consolidate the necessary individual and collective competencies of their specialty in preparation for imminent practice.^3^^,^^25^^,^^26^

Residents may work with their PDs to individualize the format and setting of the *Transition to Practice* stage to meet their goals and to achieve any needed experience and skills. Some learners at this stage, for example, may need to strengthen competencies in teaching, whereas others may have needs related to leadership, practice management, or the further sophistication of clinical skills. There may also be residents who choose to work in a clinical setting that aligns well with their chosen practice setting (e.g. a community-based hospital outside their core-training site).

Based on the above characteristics of *Transition to Practice*, we developed the following working definition:

*Transition to Practice* is the final stage of specialty residency education, acting as a bridge to autonomous practice. The consolidation of professional activities in this stage serves to increase competence and confidence for practice readiness. The format and setting are informed by the needs and goals of each learner, as well as the requisite competencies for safe and effective practice of their specialty.

Table 1 presents TTP content areas the authors identified from the literature, Royal College publications, and consensus discussion.

**Table 1:** Content for *Transition to Practice* stage

Billing Communication skills Conflict resolution Experience in high-volume environment Further sophistication of clinical skills Health advocacy How to be an effective teacher and supervisor How to get hired in your setting of choice How to set up a practice (i.e. purchasing medical equipment, hiring staff) Maintenance of Certification requirements and strategies Medico-legal aspects of practice Leadership of health care team Life-long learning strategies Opportunities for scholarship in your discipline Patient complication analysis Patient safety Physician wellness/ work–life balance strategies Procedure/ surgical skill autonomy Quality improvement Systematic approach to reflective practice Time management skills

### Survey responses

We sent the survey to 80 Program Directors (PDs) at the University of Toronto, and we received 51 responses. While this suggests a 64% response rate, some PDs, without prompting, forwarded the survey to other faculty in their program, and so the final number of survey recipients is unknown. As only one response was desired from each program, we retained PD responses, and excluded other faculty responses from the same specialty. In cases with responses from multiple faculty members but not the PD, the PD was consulted as to which one to include. Five individuals completed the first 2 questions only; these partially completed responses were excluded from the final analysis. The result was 35 completed surveys, representing 33 programs, with two respondents remaining anonymous; giving a response rate of 41% of the programs.

Table 2 presents the survey participants by clinical department. The Department of Paediatrics provided the greatest *proportion* of responses, with Surgery and Medicine being equal in *number* of responses. The Other category includes Laboratory Medicine,

Diagnostic Imaging, and Psychiatry. Appendix C in Supplemental Materials provides a complete list of programs that responded.

**Table 2:** Departments of survey participants

Department	n (N=35)	%
Pediatrics	11	33.3
Medicine	8	24.2
Surgery	8	24.2
Other *	6	18.2
Anonymous^¥^	2	0.1
Total	35	100

* Other includes: Anatomic pathology, Diagnostic radiology, Nuclear medicine, Pediatric diagnostic radiology, Psychiatry, Child and adolescent psychiatry. ¥ Two respondents remained anonymous, as they did not provide their departments or their names

## Survey results

### Important TTP content

Survey respondents selected from a list of topics that the authors had previously identified as relevant for TTP (Table 1). Table 3 illustrates their selections when they were asked to indicate all content areas they considered important. For the full cohort, their top items were *Time management skills* (82.9% of responses) and *Billing* (80% of responses). Table 4 presents the topics selected as one of the three most important, by three or all four sub-groups. However, other differences among specialties were also observed. For example, those from Pediatrics chose *How to get hired in your setting of choice* (91%) and *Leadership of health care teams* (91%) the most frequently. Those from Surgery had the greatest consensus in selecting *Billing* (100%), *Medical-legal aspects of practice* (100%), and *Communication skills* (100%). Medicine specialties had top responses of *Billing* (100%), *Medico-legal aspects* (88%), and *Experience in high-volume environment* (88%). Programs categorized as Other had top responses of *Communication skills* (100%) and *Time management skills* (83%).

**Table 3:** Content identified as important for *Transition to Practice* in full group

Topics*	Topics*	%
1. Time management skills	29	82.9
2. Billing	28	80.0
3. How to get hired in your setting of choice	27	77.1
4. Medico-legal aspects of practice	26	74.3
5. Communication skills	25	71.4
6. Experience in high-volume environment	24	68.6
7. Physician wellness/ work–life balance strategies	24	68.6
8. How to set up a practice (i.e. purchasing medical equipment, hiring staff)	23	65.7
9. Leadership of health care teams	23	65.7
10. Lifelong learning strategies	23	65.7
11. Further sophistication of clinical skills	22	62.9
12. How to be an effective teacher and supervisor	22	62.9
13. Maintenance of certification requirements and strategies	20	57.1
14. Conflict resolution	19	54.3
15. Health advocacy	19	54.3
16. Quality improvement	19	54.3
17. Systematic approach to reflective practice 18. Patient safety 19. Patient complication analysis 20. Opportunities for scholarship in your discipline 21. Procedure/ surgical skill autonomy	19 17 15 14 11	54.3 48.6 42.9 40.0 31.4

**Table 4:** Topics selected as one of top three for *Transition to Practice*

	Full group (*N* = 35)		Pediatrics (*N* = 11)		Surgery (*N =* 8)		Medicine (*N* = 8)		Other^¥^ (*N* = 6)	
	n*	**%**	***n***	**%**	***n***	**%**	***n***	**%**	***n***	**%**
Selected by all four sub-groups^**^										
Further sophistication of clinical skills	12	34.3	4	36	2	25	2	25	4	67
Time management skills	10	28.6	4	36	2	25	1	13	1	17
Experience in high-volume environment	8	22.9	2	18	1	13	2	25	3	50
Leadership of health care teams	8	22.9	3	27	2	25	1	13	2	33
Selected by three sub-groups^**^										
Billing	8	22.9	4	36	2	25	1	13	0	0
Communication skills	7	20	3	27	1	13	0	0	3	50
How to set up a practice	11	31.4	3	27	3	38	5	63	0	0
Physician wellness	7	20	2	18	1	13	3	38	0	0

* Numbers may not total across the row, as two anonymous respondents could not be assigned to a specialty.

** Sub-groups refer to the grouped specialties of Pediatrics, Surgery, Medicine, and Other

¥ Other includes: Anatomic pathology, Diagnostic radiology, Nuclear medicine, Pediatric diagnostic radiology, Psychiatry, Child and adolescent psychiatry.

### (2) Three most important items

Of the topics that each respondent had selected, they then chose the three they considered the most important. In the full group, the top three topics they selected were *Further sophistication of clinical skills* (34.4 %), *How to set up a practice (31.4 %)*, and *Time management skills (28.6 %)*. No respondent selected *Health advocacy, Opportunities for scholarship in your discipline*, or *Patient complication analysis* as one of their top three topics. Table 4 illustrates the differences in selection by subgroup.

### (3) Desired outcomes, teaching approaches, and assessment modalities

For their top three topics, respondents then identified the desired outcome (based on Miller’s Framework for Clinical Assessment), the most appropriate teaching approach out of five approaches listed, and the most appropriate assessment modality out of five modalities listed.^14^^,^^15^ The desired outcomes, most appropriate teaching approaches, and most appropriate assessment modalities, are presented in the Supplemental Materials, Appendix D, Tables A to C.

The respondents selected the top level of Miller’s triangle, *Does*, most frequently, and *Knows How* second most often. Under teaching approaches, no respondents selected the listed option of *Assigned Reading* for any topic (Supplemental Materials, Appendix D, Table B). They chose *Opportunity to Practice* for all but two items, those being *How to Get Hired* and *How to Set Up a Practice*, which were both to be covered in the academic curriculum.

Regarding assessment strategies, respondents selected *Real-life Settings* and *Feedback and Coaching* for 10 of the 12 topics, and *Guided Self-assessment and Reflection* was the next most popular, as survey participants chose it for six topics (Supplemental Materials, Appendix D, Table C). For five of the topics, respondents selected *Simulation*, while only two topics corresponded to *Written Test*. It is noteworthy that *Written Test* ranked so low, as this is consistent with the evolution of medical education, with greater emphasis on workplace-based assessments.^27^

### (4) Clinical setting and location

Twenty-seven individuals responded to the question: “For your specialty, are there specific features of the clinical setting or location that would be best suited for learning practice readiness?” Survey respondents described a total of 32 features in response to this question. The feature listed the most often was a variation on clinical practice (*n*=8), with three comments emphasizing high volume as a key feature. This was followed by community setting (*n*=7) and acute care hospital (*n*=6). Of note, three comments highlighted the feature of ensuring a diversity of settings (e.g., academic and community; clinic and operating room; inpatient and outpatient). A complete list of the comments is presented in Supplemental Materials, Appendix F.

### (5) Additional comments

Survey respondents were invited to comment on how to ensure the TTP stage prepares learners to become confident and competent practitioners. Eleven individuals responded, with no overall theme. Several comments supported trends observed from earlier questions, some of which the authors highlight in the next section of this paper. The complete comment list is available in Supplemental Materials, Appendix F.

## Discussion

This study offers unique insight into the perspectives of 33 University of Toronto specialty programs, through the responses of 35 Program Directors (or their designates) on the *Transition to Practice* stage of training, at a time when Competency by Design is being implemented. The results have practical applications for Canadian residency programs, as they too have local needs for curriculum development, implementation, and delivery.

Certain topics are seen as important for *Transition to Practice* regardless of specialty, namely *Time management* and *Billing*. Additionally, there are also features of TTP content that are highly variable depending on the specialty. For example, as shown in Table 3, 100% of respondents from Surgery and Diagnostic/Laboratory identified *Communication skills* as important content; however, pediatrics and medicine respondents selected it significantly less frequently (64% and 38%, respectively). When respondents were asked to identify their top three topics, there was only convergence among departments regarding *Time management*. The rest of the topics were specialty-dependent (Table 4). In the Top Three question, Surgery and Diagnostic/Laboratory again selected *Communication skills* more frequently than Pediatrics or Medicine. One interpretation of this trend may be that the medical specialties encounter most communication skill related competencies earlier in residency. This perspective is supported by the following comment offered by a survey respondent:

“All issues listed at the start of the survey seem important. I didn't choose all because I feel those will be covered earlier in the program.”

Another interpretation may be that the Surgical and Diagnostic/Laboratory programs are identifying deficiencies in communication skills in recent graduates. However, communication skills cover many domains, and non-surgical specialties may have equal concerns. The following survey participant comment, for example, illustrates concern regarding communication skills from a non-surgical specialty:

“Handling high pressure conversations over diagnosis and withdrawal of care are the most critical skill in my practice area and is one of the most poorly taught.”

This divergence of responses between specialties highlights that although there may be an overall shared purpose regarding the TTP stage, and a common understanding of its importance among specialties, programs will need local flexibility on the curriculum implementation side to meet the specific needs of their residents. Differences across specialties should not be ignored. For example, the timing and environment for learning communication skills for a surgical resident to lead a surgery may be different than for a medical resident learning to lead a team on the ward.

One survey respondent commented on this need for personalization:

“It will not be a one-size-fits-all solution, so the ability to be flexible to meet the demands of the individual trainee will be important for success.”

Once respondents had to narrow their choice to their top three topics, *Billing* and *Time management* were no longer as high ranking in the full cohort as when they had unlimited options. *Further sophistication of clinical skills* was chosen the most frequently, followed by *How to set up a practice*, and then *Time management*. This may reflect the reality that when autonomous practice is imminent there is a prioritization of competencies and a primary focus on new maturation responsibilities. At the same time, there appears to be a secondary purpose of TTP, which is consolidating prior skills. This supports the working definition of TTP developed by the authors in the first part of the study.

A survey respondent highlighted the area of focus of TTP:

“[*Transition to Practice*] should focus on skills that are unique to this time and not on skills that are important and taught throughout training.”

This study provides the medical education community with further practical implications for curriculum planning for the *Transition to Practice* stage, as well as for readiness to practice in other education models. For example, respondents selected the desired outcome from Miller’s Triangle of “Does,” the teaching approach of “Opportunity to Practice,” and the assessment method of “Real-life setting” most often in the survey (Tables 2 to 4). No respondent selected *Assigned reading* as a teaching activity; and they chose *Written tests* only twice for an assessment approach. With the identification of *Real-life settings, Feedback and coaching, Guided self-assessment*, and *Reflection for assessment* as important approaches, residency programs will find themselves working towards identifying feasible and systematic ways to implement direct observation, feedback delivery, and self-reflection tools.^27^ Building capacity to shape TTP with clinical settings that emulate the setting of imminent practice for learners will also be important.

As one survey participant commented:

“[TTP] should model potentially future practice...The experience should be made to reflect the most real-life practice setting as much as possible.”

Collaboration among residency programs may help in sharing learning, particularly among similar programs. Several PDs shared with the researchers that they found the flow of questions and tools provided in the survey indispensable to the future process of matching EPAs to teaching activities and assessment approaches. The process outlined by the survey lends itself well to curriculum design workshops.^16^

Interestingly, many of the comments from the survey and an international medical education conference workshop reflect and reinforce the definition developed by the authors for the TTP stage. Although the definition was provided to survey participants, it was not accessible when comments were given. As the workshop was conducted after study completion, the comments from participants were not formally included or analyzed in this paper. However, the workshop participants’ comments did help the authors during the analytic process and manuscript preparation, as they helped identify areas that were important.

Given the range of programs represented, the patterns that have emerged are certainly relevant to Program Directors from other Canadian institutions. While this study focused on the *Transition to Practice* stage, some themes (e.g. considering specialty-specific needs, prioritizing content, and identifying desired outcomes and approaches) also apply more broadly to curriculum development across the competence continuum.

## Limitations

The limitations of this study relate to being from a single institution and to those inherent to surveys. The results provide trends for further exploration rather than definitive conclusions, and may not be generalizable to other institutions. The authors of this study developed the survey, with limited item pre-testing, thus its validity and reliability are not known. Future research, such as using focus groups or observational methodology, can confirm emerging patterns suggested by the results. Although the narrative literature review component of this study had a focused search strategy, we supplemented it with multiple additional sources, including: RC publications, consensus discussion among authors, local EPA examples, and key informant review.

We did not include duplicate responses from the same program in the analysis in order to ensure that each program had an equal contribution. The Program Director response was given preference, as the aim of the study was to focus on their perspectives. A disadvantage to this approach is a reduction in sample size, which decreased the power to detect meaningful differences among respondents.

Despite the reduction of responses from 51 to 35, a wide variety of residency programs contributed, with comparable representation from pediatric, medicine, surgery, and radiology/laboratory. The full cohort, however, is predominantly non-surgical (71.4%). For this reason, subgroups were formed using departments. Given the small numbers of participants in each subgroup (ranging from six to 11), however, departmental differences may be over-estimated. Additionally, although radiology, laboratory, and psychiatry programs were categorized together, they likely have differing needs for *Transition to Practice*. The programs grouped as Medicine, Pediatrics, and Surgery are likely more similar, creating a potential for misclassification bias that is not equal among the groups.

The options related to the best teaching approaches are not mutually exclusive, and the requirement to select only one option may decrease the validity of these responses. For example, including a topic in the curriculum more than once overlaps with both inclusion in the TTP curriculum and the opportunity to practice. Similarly, assessment methods are not mutually exclusive, as feedback and coaching may occur in a simulation or a real-life setting. For this reason, these results may not definitively represent faculty preferences.

## Conclusion

As Competency by Design is implemented across Canadian residency programs, local curriculum development, implementation, and delivery will follow. Local deliberation on important content areas, teaching approaches, and assessment tools is necessary for effective implementation of the nationally approved curriculum design for *Transition to Practice*.

The results of this study provide a prototype for local curriculum development processes, as well as considerations for CBD implementation. Using a working definition for TTP is helpful. The definition provided may be useful during curriculum development, as reflected in comments from Program Directors across a range of specialties. Certain TTP content areas appear to be important for inclusion in teaching, and assessment across specialties, specifically: *Further sophistication of clinical skills, How to set up a practice*, and *Time management skills*. In addition, the needs of learners appear to be highly specialty-specific; thus it is recommended that serious consideration of maturation responsibilities for residents of the specialty be undertaken when developing TTP curricula. Ensuring TTP occurs in a clinical setting that emulates the learner’s imminent practice is important. This has implications for operationalizing workplace-based learning, teaching, and assessment. The process used in the survey to identify *Desired Outcome, Teaching Approach*, and *Assessment Approach* may be adapted for use in curriculum development across Canada and potentially in other countries. As practice readiness is relevant to all residency programs, regardless of education model, the process and results of this study may be helpful in a diversity of contexts.

## Footnotes

a The medical education community will benefit from abandoning the term Junior Attending, used in the previous medical education model. Regulatory authorities’ views, such as the College of Physicians and Surgeons of Ontario (CPSO) policy on Professional Responsibilities in Postgraduate Medical Education^11^, emphasize the importance of learners stating their stage in the program to patients. The designation of Junior Attending is misleading, as learners who have yet to receive their independent practice certification imply that they are attending physicians.
