# Supplementary material for: Practical solutions for implementation of Transition to Practice curricula in a competency-based medical education model
Source: Can Med Educ J. 2020 Aug 6;11(4):e39–50. doi: 10.36834/cmej.67821 (PMC7417824; doi:10.36834/cmej.67821)
Supplement: Supplementary file 1 — Supplementary Tables [file CMEJ-11-e039-s001.pdf]

# Appendix A: Survey questions



## Default Question Block

*Dear Colleague*

*You are being asked to complete this survey in order to share your perspective on what is important content for the Transition to Practice stage of medical education for your specialty.*

*To help you answer the questions, please use the following working definition of Transition to Practice, based on the literature, expert opinion and Royal College publications.*

> ***Transition to Practice** is the final stage of specialty education, acting as a bridge to autonomous practice. The consolidation of professional activities in this stage serves to increase competence and confidence for practice readiness.*
> *The format and setting is informed by the needs and goals of each learner, as well*
> *as the requisite competencies for safe and effective practice in their specialty.*

***Participation in this survey is completely voluntary.** Responses will be viewed in aggregate form, and cannot be linked back to any individual. This survey will be used for research and program evaluation purposes only. Your participation will be equated as consent for your responses to be used in the manner described above.*

*Thank you.*

*Layli Sanaee, MD, FRCPC*
*Emergency Physician, University Health Network*
*Lecturer, Department of Medicine, University of Toronto*

*Susan Glover Takahashi MA(Ed),PhD*
*Director, Education & Research*
*Lead, Education Integration Group – CBME*
*Post MD Education – Postgraduate Medical Education*

## Block 1

What is your name?

[ ]

What is your program?

[ ▾ ]

Below is a list of topics that have been identified as important to consider in the *Transition to Practice* stage.

For your specialty, please identify all that you think are important:

- ☐ Billing
- ☐ Communication skills
- ☐ Conflict resolution
- ☐ Experience in high-volume environment
- ☐ Further sophistication of clinical skills
- ☐ Health advocacy
- ☐ How to be an effective teacher and supervisor
- ☐ How to get hired in your setting of choice
- ☐ How to set up a practice (i.e. purchasing medical equipment, hiring staff)
- ☐ Maintenance of Certification requirements and strategies
- ☐ Medico-legal aspects of practice

- ☐ Leadership of health care team
- ☐ Life-long learning strategies
- ☐ Opportunities for scholarship in your discipline
- ☐ Patient complication analysis
- ☐ Patient safety
- ☐ Physician wellness/ work-life balance strategies
- ☐ Procedure/ surgical skill autonomy
- ☐ Quality improvement
- ☐ Systematic approach to reflective practice
- ☐ Time management skills
- ☐ Other (please specify): ____________

Of the following topics that you identified as important, which are the top three most important?

- ☐ » Billing
- ☐ » Communication skills
- ☐ » Conflict resolution
- ☐ » Experience in high-volume environment
- ☐ » Further sophistication of clinical skills
- ☐ » Health advocacy
- ☐ » How to be an effective teacher and supervisor
- ☐ » How to get hired in your setting of choice
- ☐ » How to set up a practice (i.e. purchasing medical equipment, hiring staff)
- ☐ » Maintenance of Certification requirements and strategies
- ☐ » Medico-legal aspects of practice
- ☐ » Leadership of health care team
- ☐ » Life-long learning strategies
- ☐ » Opportunities for scholarship in your discipline
- ☐ » Patient complication analysis
- ☐ » Patient safety
- ☐ » Physician wellness/ work-life balance strategies
- ☐ » Procedure/ surgical skill autonomy

- [ ] » Quality improvement
- [ ] » Systematic approach to reflective practice
- [ ] » Time management skills
- [ ] » Other (please specify):

## Block 3

For your selected priority topic of **${lm://Field/1} ${q://QID12/ChoiceTextEntryValue/x22}**, please indicate the option that best identifies the DESIRED OUTCOME:

- ○ 1. Orientation
- ○ 2. Knows
- ○ 3. Knows How
- ○ 4. Shows
- ○ 5. Does

For your selected priority topic of **${lm://Field/1}**, please indicate the option that best identifies the most appropriate TEACHING APPROACH:

- ○ Assigned reading
- ○ In TTP Curriculum
- ○ In Curriculum more than once
- ○ Opportunity to practice in simulated situation
- ○ Opportunity to practice

For your selected priority topic of **${lm://Field/1}**, please indicate the option that best identifies the most appropriate ASSESSMENT approach:

- ○ Guided self-assessment and reflection
- ○ Feedback and coaching
- ○ Written test
- ○

Assessed in simulated situation

○ Assessed in real-life setting

## Block 2

For your specialty, are there specific features of the clinical setting or location that would be best suited for learning practice readiness? *Please describe.*

Do you have any comments, questions or further considerations related to ensuring that the *Transition to Practice* stage prepares learners to become confident and competent autonomous practitioners in your specialty? *Please describe*

Powered by Qualtrics

## Appendix B: Guide to curriculum design, learning, teaching, and assessment [a]

| Level | Desired Outcomes[b] | Approaches to Learning and Teaching | Approaches to Assessment[1] |
|---|---|---|---|
| 0 | Not included | • Not explicitly included. (i.e. May be prerequisite, not a priority in program, self-taught, etc.) | • Not explicitly included<br>• Formative feedback<br>• Self-assessment |
| 1 | Orientation | • Assigned reading<br>And/Or<br>• Brief overview of topic/program | • Reflection[c], self-report[d], self-assessment[e], formative feedback<br>• Not specifically assessed formatively[f] or summatively[g] |
| 2 | Knows | • Assigned reading<br>And/Or<br>• In curriculum | • Reflection, self-report, self-assessment, formative feedback<br>And<br>• Via written assignments, written tests, verbal tests, case reports |
| 3 | Knows how | • Assigned reading<br>And/Or<br>• In curriculum | • Reflection, self-report, self-assessment<br>And<br>• Via written assignments, written tests, verbal tests, case reports |
| 4 | Shows | • Assigned reading<br>And/Or<br>• In curriculum, often more than once<br>And/Or<br>• Opportunity to practice | • Reflection, self-report, self-assessment<br>And<br>• Assessed in a simulated situation (i.e. written simulation, clinical simulation, lab setting) |
| 5 | Does | • Assigned reading<br>And/Or<br>• In curriculum, often more than once<br>And/Or<br>• Opportunity to practice in simulated situations | • Assessed in a "real-life" setting (e.g. encounter forms, mini-CEx) |

[a] With permission: Glover Takahashi S. A "Top Line" Guide to Curriculum Design, Learning, Teaching and Assessment. 2012. Updated 2017. © Glover Takahashi 2012, updated September 2017.
[b] Adapted from Miller, G. E. (1990). The assessment of clinical skills/competence/performance. *Academic Medicine,65,* S63–S67.
[c] **Reflection** means process of learning from experience. The learning may result in changes to practice. It involves mastery of a subject plus active analysis of the purposes and consequences of decision-making (Paschal, Jensen and Mostrom, 2002).
[d] **Self-report** means factual reports on information of interest (Glover Takahashi, McIlroy and Beggs, 2008).
[e] **Self-assessment** means an activity and process by which an individual describes and judges the nature, extent, and level of their own learning or performance; self-assessment is a component of reflection and is often a part of, or an outcome of, a prior learning assessment process.
[f] **Formative assessments** are assessments done for purposes of developing competence and generally include feedback on performance and commentary on opportunities for improved performance.
[g] **Summative assessments** are assessments done for purposes of providing a summary conclusion or judgment about the competence of the individual. May or may not include feedback on performance and commentary on opportunities for improved performance.

## Appendix C: Programs of survey participants

| Programs (*N* = 35*) |
|---|
| Adolescent medicine |
| Anatomical pathology |
| Anesthesiology |
| Cardiology – adult |
| Cardiology – pediatric |
| Child and adolescent psychiatry |
| Clinical immunology and allergy – pediatric |
| Colorectal surgery |
| Critical care medicine – adult |
| Critical care medicine – pediatric |
| Dermatology |
| Diagnostic radiology |
| Diagnostic radiology – pediatric |
| Emergency medicine |
| Emergency medicine – pediatric |
| Endocrinology and metabolism – adult |
| Endocrinology and metabolism – pediatric |
| General internal medicine |
| General surgery |
| Infectious diseases – pediatric |
| Medical genetics |
| Medical oncology |
| Neurosurgery |
| Nuclear medicine |
| Obstetrics and gynecology |
| Orthopedic surgery |
| Otolaryngology |
| Pain medicine |
| Pediatrics – general |
| Plastic surgery |
| Psychiatry |
| Rheumatology – pediatric |
| Vascular surgery |

** 2 survey respondents remained anonymous as they did not provide their department, thus 33 programs are listed*

## Appendix D: Detailed results

### Table A: Desired outcomes for topics selected as one of the three most important

| Topic | N* | Desired Outcome | DO**/ N (%) |
|---|---|---|---|
| Further sophistication of clinical skills | 12 | Does | 10/12 (83) |
| How to set up a practice | 11 | Knows how | 6/11 (55) |
| Time management skills | 10 | Does | 6/10 (67) |
| Billing | 8 | Does<br>Knows how | 3/8 (43)<br>2/8 (29) |
| Experience in high-volume environment | 8 | Does | 6/8 (75) |
| Leadership of health care team | 8 | Does | 6/8 (75) |
| Communication skills | 7 | Does | 7/7 (100) |
| How to get hired in your setting of choice | 6 | Knows how | 3/6 (50) |

** N= number of respondents who selected the indicated topic as One of Three Most Important; only topics selected by ≥6 are shown*
*** DO= number of respondents who selected the indicated Desired Outcome for the respective topic; only Desired Outcome(s) selected by ≥ 50% per topic are shown*
*For example, 12 respondents selected Further sophistication of clinical skills as one of Three Most Important topics; and 10/12 respondents selected Does as Desired Outcome*

### Table B: Best teaching approach for the topics selected as one of the three most important

| Topic | *N** | Best Teaching Approach | DO**/*N*(%) |
|---|---|---|---|
| Further sophistication of clinical skills | 12 | Opportunity to practice | 10/12 (83) |
| How to set up a practice | 11 | In TTP curriculum<br>More than once | 5/11 (46)<br>4/11 (36) |
| Time management skills | 10 | Opportunity to practice | 6/10 (67) |
| Billing | 8 | Opportunity to practice | 6/8 (86) |
| Experience in high-volume environment | 8 | Opportunity to practice | 7/8 (87.5) |
| Leadership of health care team | 8 | Opportunity to practice | 8/8 (100) |
| Communication skills | 7 | Opportunity to practice | 6/8 (86) |
| How to get hired in your setting of choice | 6 | In TTP curriculum | 3/6 (50) |

* *N*= number of respondents who selected the indicated topic as One of Three Most Important; only topics selected by ≥6 are shown
** DO= number of respondents who selected the indicated Best Teaching Approach for the respective topic; only Desired Outcome(s) selected by ≥ 50% per topic are shown
*For example, 12 respondents selected Further sophistication of clinical skills as one of Three Most Important topics; and 10/12 respondents selected Opportunity to practice as best teaching approach*

### Table C: Appropriate Assessment Approach for Topics Selected as One of the Three Most Important*

| Topic | *N** | Assessment method | DO**/N(%) |
|---|---|---|---|
| Further sophistication of clinical skills | 12 | Real-life settings | 10/12 (83.3) |
| How to set up a practice | 11 | Guided self-assessment and reflection | 6/11 (54.5) |
| Time management skills | 10 | Real-life settings<br>Feedback and coaching<br>Guided self-assessment and reflection | 4/10 (40)<br>2/10 (20)<br>2/10 (20) |
| Billing | 8 | Real-life settings<br>Feedback and coaching | 3/8 (42.9)<br>2/8 (29) |
| Experience in high-volume environment | 8 | Real-life settings | 8/8 (100) |
| Leadership of health care team | 8 | Real-life settings | 5/8 (62.5) |
| Communication skills | 7 | Real-life settings | 6/7 (85.7) |
| How to get hired in your setting of choice | 6 | Guided self-assessment and reflection | 3/6 (50) |

* *N*= number of respondents who selected indicated topic as One of Three Most Important; only topics selected by ≥6 are shown
** DO= number of respondents who selected indicated Assessment method for respective topic; only Desired outcome(s) selected by ≥ 50% per topic are shown
*For example, 12 respondents selected Further sophistication of clinical skills as one of Three Most Important topics; and 10/12 respondents selected Real-life settings as most appropriate assessment method*

## Appendix E: Features of clinical settings or location for learning practice readiness

Clinical Practice (*N* = 8)

- Any busy clinical practice
- Attendance and participation in surgeon's private office settings
- Clinic
- High-volume clinical practice; varied settings include clinic and OR
- Hospital consult service and outpatient clinics
- Ideally a senior clinical rotation in the same subspecialty that the trainee will do in fellowship/in practice (if fellowship does not occur)
- In the clinical environment
- The high volume and resident supervision

Community (*N* = 6)

- Academic settings and community to provide contrast and range of realities
- Community
- Community setting (where they will be practicing) makes sense to be the setting to learn leadership/manager skills for their future practice
- Generally, the academic setting, but for those who choose a community setting it would be important to increase this exposure beyond what is normally provided
- Most train and then practice in academic hospital setting, but for those who choose a predominantly community setting we need to provide more exposure and focus on added skills, such as billing
- Usually outpatient setting in the community

Acute Hospital (*N* = 6)

- Acute inpatient
- Acute neonatal
- Inpatient
- Bedside in the emergency department
- High-volume clinical practice; varied settings include clinic and OR
- Operating room

AHD (*N* = 2)

- Academic half-day
- In the classroom (didactic)

Longitudinal Clinic (*N* = 2)

- Longitudinal clinic
- Our new longitudinal ambulatory experience (across all 5 years) allows refinement of manager/leader as well as teaching and clinical competence that approximates real world practice

Miscellaneous (*N* = 9)

- Access to audiology services
- Supervision of cardiac stress test
- Exposure to a variety of different practice contexts
- Imaging
- Reading and reporting medical imaging
- Multidisciplinary clinics and rounds that would help to teach and observe learners in collaborator and communicator roles
- Simulated setting
- Simulated sign-out of cases would work well

Did Not Understand the Question (*N* = 2)

- At the completion of training, perhaps in June after certification examinations at the university
- I am not sure I understand the question. If you are asking what features of the training environment are best for learning the skill of practice readiness, the answer is that it must be a real, rather than simulated environment.

## Appendix F Additional comments

| |
|---|
| Some of these parameters are hard to assess as it is in real life |
| All issues listed at the start of the survey seem important. I didn't choose all because I feel those will be covered earlier in the program. |
| Will need to have a way to ensure that these topics get covered in AHD or in the community clinical setting, |
| Handling high-pressure conversations over diagnosis and withdrawal of care are the most critical skill in my practice area and is one of the most poorly taught. |
| Demonstration of required outcome is of most value |
| Often this is really up to the resident--the opportunities and the expectations are there for this transition stage in our program, however, not all residents engage in this opportunity. The timing of the Royal College Board Exams is a major issue in the last 4-6 months of the PGY5 year, with many residents focusing on the exams and not on their transition to practice. |
| This is an important aspect that is not well addressed in current training - we can do better. It will not be a one-size fits all solution, so the ability to be flexible to meet the demands of the individual trainee will be important for success. |
| It should focus on skills that are unique to this time and not on skills that are important and taught throughout training. Many of the topics listed in the first question are topics that are very much part of training and do not need specific focus at this time in training. |
| Infuse the culture of life-long learning and curiosity |
| Should model potentially future practice. Limitation of TTP block rotations are the trainees' multiple half day backs away from the inpatient unit which makes the experience somewhat artificial. The experience should be made to reflect the most real-life practice setting as much as possible |
| Important and useful concept. There needs to be recognition that many trainees do fellowships and that their actual practice start may be significantly delayed from date of graduation |
